# Variable toxicity of inorganic mercury compounds to *Artemia* elicited by coexposure with dissolved organic matter

**DOI:** 10.1007/s11356-024-35558-y

**Published:** 2024-11-21

**Authors:** Christoph Gade, Rebecca von Hellfeld, Lenka Mbadugha, Graeme Paton

**Affiliations:** 1 National Decommissioning Centre, University of Aberdeen, Aberdeen, Scotland UK; 2 School of Biological Sciences, University of Aberdeen, Cruickshank Building, St. Machar Drive, Aberdeen, Scotland AB24 3UU UK

**Keywords:** Marine environment, Ecotoxicology, Hatching assay, Bioaccumulation

## Abstract

The chemical behavior of mercury (Hg) and its interactions with naturally occurring ligands shape its environmental fate and impact. The neurotoxic properties of Hg are widely known and studied both in vitro and in vivo*.* However, there continues to be limited information on the influence of chelation with large organic ligands on the toxicity to marine macro-organisms. This work examined the effect of Hg complexed with various types of dissolved organic matter (DOM) on the mortality and hatching success of *Artemia* sp. nauplii under varying marine media conditions. The results confirmed both, an alleviating as well as additive, DOM-specific, effect on mortality. DOM coexposure resulted in a compound specific decreased or increased toxicity in comparison with single exposure in artificial seawater, with LC_50_ values ranging from 2.11 to 62.89 µM. Hatching success under conditions of Hg exposure was almost two orders of magnitude more sensitive than toxicity in hatched individuals. Elevated DOM concentrations had no statistically significant impact on hatching success with computed EC_50_ values ranging from 196 to 324 nM.

**Graphical abstract:**

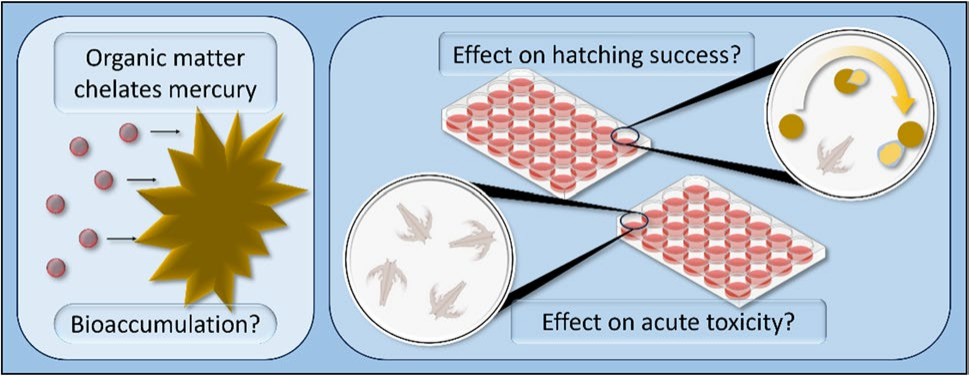

## Introduction

The marine biome is exposed to a multitude of stressors including chemical pollution. Mercury (Hg) is a ubiquitous heavy metal with intrinsic elemental characteristics that allow it to speciate into different Hg compounds with diverse physicochemical properties (Gworek et al. 2016, 2020). In the context of this manuscript, speciation is defined as ‘the distribution of the element among various chemical forms which together make up the total concentration of the element in the system’ (Blust et al. 1995). The toxicity of Hg is caused by non-specific denaturing of proteins, resulting in enzyme inhibition and genotoxicity on a cellular level and structural damages on a tissue level (Krupp et al. 2016), as well as a resource depletion of detoxification and defence mechanisms (von Hellfeld et al. 2023a). Marine environments act as sink and source for environmental Hg with major influx from wet deposition and anthropogenic emissions and efflux primarily as surface gas evasion and sediment burial (Mason and Fitzgerald 1996). The environmental fate and transport of Hg in aquatic systems is linked with particulate and dissolved organic matter (DOM), which chelate dissolved Hg with highly reactive functional groups (Dyrssen and Wedborg 1991; Gerbig et al. 2011; Jeremiason et al. 2015; Gade et al. 2024). Conte et al. (2007) defined DOM as ‘supramolecular aggregates of small heterogeneous molecules strongly associated by dispersive forces in apparently large molecular sizes.’ In marine environments, DOM serves as a nutrient source (Baylor and Sutcliffe 1963), drives photochemical processes (Zhou et al. 2020), and constitutes a major part of the planets’ carbon cycle (Ogawa and Tanoue 2003). Mercury is highly thiophilic, which promotes strong binding with sulfur containing heteroatomic functional groups of DOM, such as aromatic thiols (Li et al. 2019). The effect of DOM complexation on toxicity and bioaccumulation in freshwater systems is well described; however, a similar understanding for marine organisms is currently lacking (Barber-Lluch et al. 2023).

The euryhaline crustacean *Artemia* has been used in ecotoxicological testing due to several advantageous properties, such as rapid hatching, easy accessibility, and sensitivity to toxic substances (Nunes et al. 2006). Sensitive endpoints, including hatching success, growth, acute mortality, and behavioral and biomarker responses, are commonly used as evaluation criteria in *Artemia* toxicity testing (Libralato et al. 2016). *Artemia* also have the advantage of being cost-efficient and readily available as nauplii can be hatched directly from cysts without the need for culture maintenance. Although, their adaptability to diversified testing conditions (i.e., pH and salinity resistance) makes them an ideal candidate for toxicity studies, the heterogeneous genomics of “wildtype” *Artemia* have so far precluded the organism from being widely adopted in standardized testing (Libralato et al. 2016).

While the impact of water parameters including temperature, water hardness, and synthetic organic ligands on the toxicity of heavy metals such as cadmium has been studied (Blust et al. 1995; Penttinen et al. 1998; Heugens et al. 2003), a similarly comprehensive appraisal of the effects of DOM-Hg-coexposure is still lacking. This is in part due to the spatiotemporal heterogeneity of naturally occurring DOM resulting in a broad range of binding coefficients and subsequent degree of bioavailability (Ravichandran 2004). The aim of this study was to evaluate the impact of three commercially available types of DOM on the toxicity of Hg in *Artemia* based acute toxicity and hatching assays. The hypothesis that DOM would alleviate toxicity was based on previous works in this field (e.g., Penttinen et al. 1998).

## Materials and methods

Mercuric chloride (HgCl_2_, ACS grade) and mercuric acetate (HgOAc_2_, ACS grade) were purchased from Sigma-Aldrich. Three humic acids (technical grade) were purchased from Sigma-Aldrich (Prod. Nr. 53680), Thermo Fisher Scientific (Cat. Nr. 041747.06), and Carl Roth (Art. No. 7821.1). Humic acid sodium salt (Prod. Nr. H16752, technical grade) was purchased from Sigma-Aldrich. Bladderwrack powder certified reference material (NIST B2164) was purchased from the EU Joint Research Centre. Sodium chloride (NaCl, analytical grade), magnesium sulphate (MgSO_4_, analytical grade), calcium chloride (CaCl_2_ 2H_2_O, analytical grade), potassium chloride (KCl, analytical grade), and magnesium chloride (MgCl_2_ 7H_2_O, analytical grade) were obtained from Fisher Scientific. *Artemia* sp. cysts were procured from Blades Biological Ltd. (UK), subsampled, and stored in darkness at − 20 °C until further use, as recommended by other suppliers.

### DOM characterization

To allow for a higher reproducibility, commercially available types of DOM were used to make up DOM-enriched Hg stock solutions and exposure media. To further analyze the properties of individual DOM materials, elemental and Fourier-transformed infrared (FTIR) analyses were carried out before the ecotoxicological studies commenced. Preliminary toxicity tests using DOM-enriched media revealed a 20–30% toxicity elicited by the humic acids purchased from Sigma-Aldrich and Carl Roth. These compounds were subsequently excluded from any further experiments. The remaining compounds, i.e., humic acid sodium salt (Sigma-Aldrich), Bladderwrack powder (NIST B2164), and humic acid (Thermo Scientific), did not induce any observable toxic effects and are from here on referred to as DOM#1, DOM#2, and DOM#3, respectively.

Elemental analysis of DOM materials was carried out on a flash analyzer for total carbon and nitrogen (CE Instruments NA 2500 Series, UK) and a radiofrequency induction analyzer for total sulfur content (LECO CS744, US). DOM#2 was used as a reference material in all analyses. Recovery was 104.25% and 95.83% for carbon and nitrogen, respectively, and 81.66% for sulfur with a relative standard deviation of 0.48%, 0.51%, and 0.76%, respectively. Fourier transformed infrared (FTIR) spectroscopy was used to investigate the nature and abundance of functional groups in the used materials. Representative portions of the samples were transferred onto the sample area of a single reflection diamond attenuated total reflectance accessory, fitted with a ZnSe ATR crystal. The infrared spectra were recorded using a Bruker Vertex 70 Fourier transform infrared spectrometer.

### Preparation of Hg species/solutions

Artificial seawater (ASW) was prepared according to Cold Spring Harbor Protocols (2012). Saline DOM solutions were prepared in ASW with an initial concentration of 0.5 g/l and stored in complete darkness at room temperature for 8 weeks, which exceeded previously reported equilibration times (Gai et al. 2016). This was done due to the extremely low solubility of the substances in ASW. Prepared DOM media were vacuum filtered through 0.45-µm membrane filters as the dissolved alginate in the DOM#2 solution impeded filtration through 0.22-µm filters. In this study, the operational definition for DOM as ‘organic material passing a filter’ was adopted (Ogawa and Tanoue 2003). After filtration, dissolved organic carbon concentrations were determined photometrically, as the salinity of the media adversely affected total organic carbon (TOC) analysis. Briefly, DOM standard solutions were produced in deionized water and their concentration verified using a TOC analyzer (Aurora 1030W, Xylem Inc., USA). A photometric standard curve was produced from these solutions, and the saline samples assessed using this curve. Mercury stock solutions were prepared at concentrations of 10 mg/l and 1 g/l in ASW or DOM-medium for the hatching and acute toxicity assays, respectively. Stock solutions were aged for at least 4 days in darkness at 4 °C before use to facilitate Hg-DOM chelation. Exposure media were prepared fresh with 24 h (h) aerated and pH = 8.1 adjusted ASW and filtered DOM-solutions before every replicate by spiking with matrix-matched stock solution.

### *Artemia* toxicity assays

To obtain Instar I nauplii, *Artemia* sp*.* cysts were incubated in 500 ml ASW of 32.8 g/l salinity, at 25 °C, pH 8.1 ± 0.5, constant aeration (50 l/h), and constant illumination (1600 lumens) for 24 h until they hatched (Sorgeloos et al. 1978). The temperature of the hatchery (JBL, Germany) and exposure plates was kept at 25 °C throughout the experiment and only nauplii from the same generation were applied for each experiment. For the acute toxicity assay, all the wells in a 24-well plates (Greiner Bio-One, Austria) were filled with 2 ml of exposure medium and 10 nauplii were placed in each well using a pipette. On each plate, 5 columns (4 wells each) contained exposure medium with different concentrations of Hg while the remaining 4 wells were filled with matrix matched unspiked medium, serving as an internal negative control. In total, *n* = 40 organisms were exposed to each concentration per replicate. The plates were incubated in complete darkness at 25 °C for 24 h, after which the dead nauplii were counted. Here, death was defined as complete quiescence in accordance with Corner and Sparrow (1957). The assay would be considered valid if mortality in the control did not exceed 10%. A positive control was not used as the impact of media composition was assumed to also change the positive control toxicity (Kalčíková et al. 2012; Deese et al. 2016). Test solution concentration was monitored stochastically using a direct mercury analyzer (DMA-80evo, Milestone, USA). Concentrations stayed within 97–103% of assumed value (data not shown). All assays were conducted in independent triplicate.

Exposure of unhatched cysts in the hatching assay followed the above-described acute toxicity assay protocol. Instead of Instar I nauplii, 10 *Artemia* cysts were placed in each well of 24-well plates with the same concentration/negative control layout as described above. Hatching was defined as the ‘release of a free-swimming larval stage organisms upon rupture of the hatching membrane’ (Go et al. 1990). After 24 h incubation at 25 °C with constant illumination (1600 lumens), plates were checked for free swimming larvae using a stereomicroscope. In total, *n* = 40 organisms were exposed to each concentration. All assays were conducted in independent triplicate. Failure to hatch as a result of Hg exposure does not translate to mortality as embryonic development may continue inside the cuticle (Go et al. 1990). As cyst mortality was not further verified, data were expressed as EC_50_ rather than LC_50_ values.

### *Artemia* bioaccumulation and adsorption assay

To offer additional insight, a simple bioaccumulation and adsorption assay was devised with the hypothesis that toxicity values would correlate with body burden. *Artemia* sp. were hatched according to the above-described protocol and pre-concentrated (i.e., filtered through a 0.15-mm mesh screen and resuspended in a minimal amount of ASW). Approximately 1-ml aliquots of pre-concentrated suspension were dispensed onto 70-µm mesh nylon cell strainers (Falcon, USA) and the water removed by gently applying a paper towel to the bottom of the mesh. Concurrently, 3 cells within 6-well plates (Greiner Bio-One, Austria) were filled with 5 ml of exposure medium (10 mg/l Hg). Strainers with live *Artemia* were immersed in 5-ml exposure medium for 1, 2, 4, and 8 h for the bioaccumulation assay. Upon retrieval at the stipulated time points, Artemia nauplii were immersed in 70% ethanol (analytical grade, Thermo Fisher Scientific) to sacrifice and to halt bioaccumulation processes. To control for organism surface adsorption the procedure was repeated however, *Artemia* nauplii were first immersed in 70% ethanol before being exposed in the respective media. Both assays were conducted in independent triplicate. Following both assays, *Artemia* were washed thoroughly with deionized water and fresh ethanol before being scraped into 10-ml glass bottles and lyophilized overnight at − 55 °C using a Modulyo 4 K Freeze Dryer (Edwards High Vacuum International, UK). Freeze dried *Artemia* were analyzed for total Hg in triplicate using the DMA. A certified reference material (fish muscle ERM MBB422) was run every 10 samples. Limit of detection (LOD) was 0.001 ng, and recovery was 99.3% with a relative standard deviation of 3.1%.

### Statistics

All data are given as mean ± standard error unless otherwise indicated. LC_50_ and EC_50_ values in the acute toxicity and hatching assay were computed using four-parameter logistic curve fitting (Eq. 1).

1 $$\mathrm{y}=\mathrm{min}+\frac{\left(\mathrm{max}-\mathrm{min}\right)}{1+{\left(\frac{\mathrm{x}}{\mathrm{C}}\right)}^{-\mathrm{Hill slope}}}$$

In Eq. 1, ‘min’ denotes the bottom of the curve; ‘max’ denotes the top of the curve; *x* and *y* are the exposure medium concentrations and mortality, respectively; *C* defines the LC_50_ and EC_50_ concentrations, and ‘Hill slope’ characterizes the slope of the curve at its midpoint (Systat Software 2020).

Computed LC_50_ and EC_50_ values were compared using ANOVA on ranks (Kruskal–Wallis *H* test), with a Dunnett’s post hoc test. Probability values (*p*) lower than 0.05 were deemed to be statistically significant and were marked with * for *p* < 0.05, ** for *p* < 0.005, and *** for *p* < 0.001.

Bioaccumulation data were assessed using the one-compartment model developed by Janssen et al. (1991):

2 $${C}_{\mathrm{I}}(t)={C}_{\mathrm{I}0}+{C}_{\mathrm{E}}\frac{{k}_{\mathrm{A}}}{{k}_{\mathrm{E}}}\left(1-{e}^{{-k}_{\mathrm{E}}t}\right)$$

where *C*_I_ is the internal concentration; *C*_I0_ is the initial metal body burden; *k*_A_ and *k*_E_ are accumulation and excretion rates, respectively; and *t* is the time. Comparison of body burden concentrations at time intervals was done using the Kruskal–Wallis *H* test. Obtained adsorption data were fitted with a pseudo-second-order (II) kinetic model. The linear form is given as follows:

3 $$\frac{1}{{q}_{t}}=\left(\frac{1}{{k}_{2}{{q}_{e}}^{2}}\right)\times \left(\frac{1}{t}\right)+\frac{1}{{q}_{e}}$$

where *q*_*t*_ is the concentration of adsorbate on the adsorbant at time point *t*, *q*_e_ is the adsorbant saturation at equilibrium, and *k*_2_ is the pseudo-second-order rate constant (Robati 2013).

## Results

### Organic matter characterization

Both DOM#2 and DOM#3 were comparably high in total carbon and nitrogen content (33.7 and 47.4%, respectively); however, the sulfur content was markedly different with DOM#2 having the highest content out of all three organic materials (Table 1). DOM#1 was measured at < 10% total carbon, < 0.2% total nitrogen, and below LOD total sulfur content which is very low in comparison with the other materials.

**Table 1:** Elemental composition of three dissolved organic carbon materials. Values are means ± standard error (*n* = 3)

Carbon source	DOM#1	DOM#2	DOM#3
C (%)	9.15 ± 0.69	33.67 ± 0.29	47.42 ± 0.09
N (%)	0.13 ± 0.05	1.25 ± 0.02	1.00 ± 0.05
S (%)	< LOD	2.29 ± 0.06	0.02 ± 0.02

**Fig. 1:**
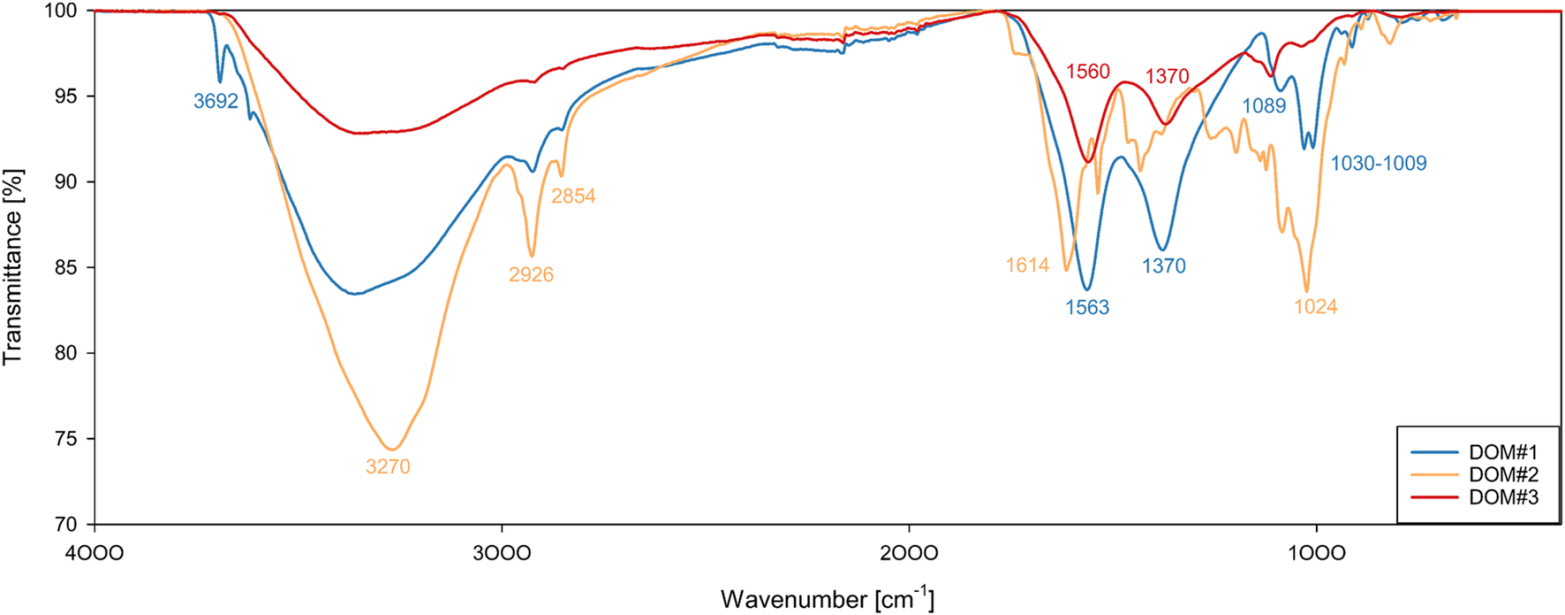
FTIR spectra of three types of organic matter. Raw absorbance spectra data were transformed to transmittance data and normalized against 100% transmittance

The FTIR spectra of the humic acids exhibited similar general characteristics such as the broad absorption band ranging from 3400 to 3200 cm^−1^ due to O–H stretching of phenol and alcohol groups, sharp peaks around 2920 and 2850 cm^−1^ in varying intensities (DOM#2 > DOM#1 > DOM#3) attributed to asymmetric and symmetric stretching of aliphatic bonds (CH_2_ and CH_3_ respectively), and several peaks spanning 1600–1000 cm^−1^ indicating a variety of functional groups (Fig. 1). After media preparation and filtration, dissolved organic carbon (DOC) concentrations in the three DOM-rich media were determined to be 3.31, 5.07, and 14.29 mg/l for DOM#2, DOM#3, and DOM#1, respectively.

### Acute toxicity and hatching assays

**Fig. 2:**
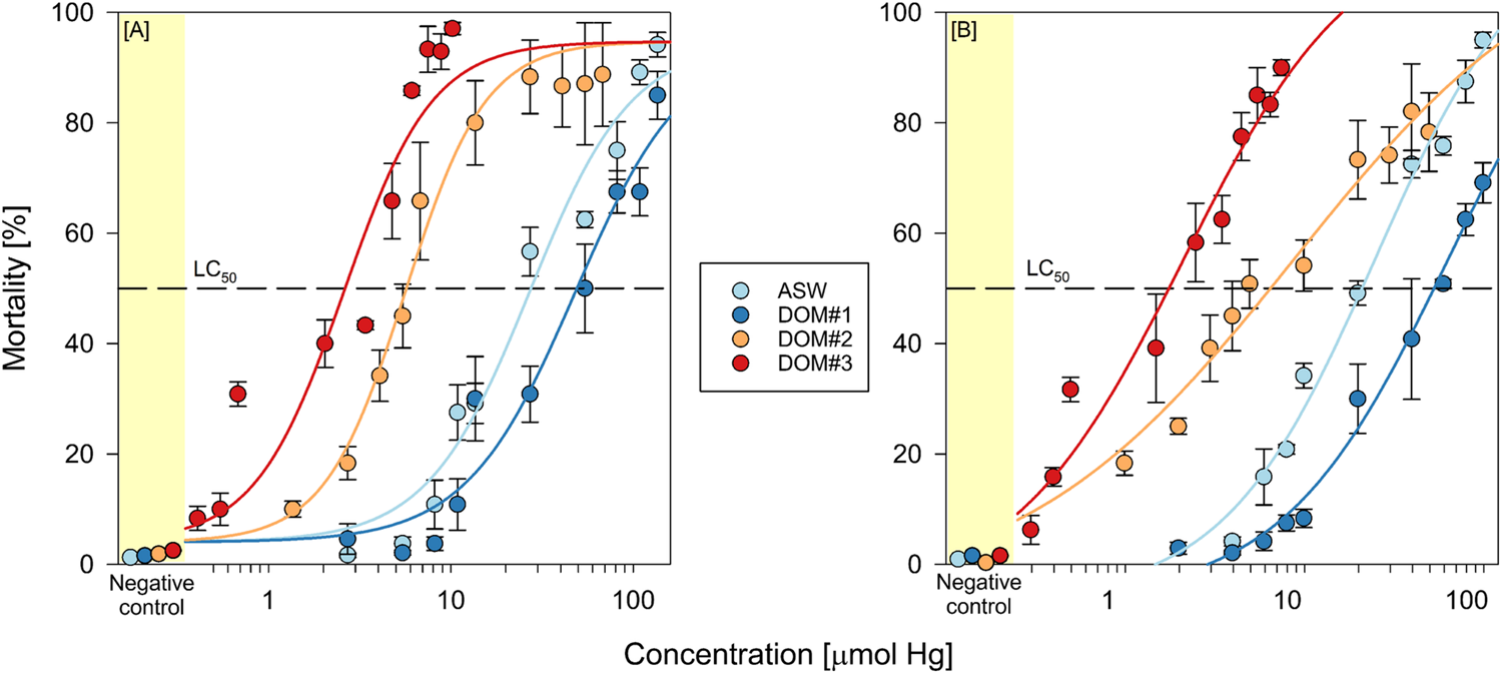
Lethal effects in Instar I *Artemia* sp*.* after 24 h coexposure to **A** HgCl_2_ and **B** HgOAc_2_ with DOM#1–3 media (see respective colors). Assays were conducted in independent triplicate, solid lines show the combined four parameter logistic fit, and error bars show the standard error of the mean

Exposure of *Artemia* nauplii in unspiked media did not result in statistically different background mortalities between the different media (*p* = 0.715). The mortality recorded in the negative controls of the acute toxicity setup was 1.09 ± 0.04, 1.56 ± 0.05, 1.09 ± 0.05, and 2.03 ± 0.06% for ASW and DOM#1–3, respectively. Single and coexposure of Instar I *Artemia* sp. nauplii with HgCl_2_ and HgOAc_2_ in ASW (single exposure) and DOM-rich media (coexposure) caused dose-dependent increases in mortality rates relative to the negative control (Fig. 2). There was no observable precipitation of HgCl_2_ and HgOAc_2_ within the used concentration ranges in pure ASW. However, a precipitation of red solids was observed during the preparation of DOM-rich stock solutions. This precipitate was not further investigated and did not seem to permanently exist in solution. Single and coexposure of *Artemia sp.* cysts with HgCl_2_ and HgOAc_2_ in ASW (single exposure) and DOM-rich media (coexposure) caused dose-dependent increases in mortality rates relative to the negative control (Fig. 2). Computed LC_50_ values derived from DOM coexposure were compared against those derived from single exposure in ASW. For coexposure with HgCl_2_ and HgOAc_2_, LC_50_ values were significantly lower in coexposure with DOM#2 and DOM#3 in comparison with single exposure in ASW. In the case of DOM#1, the increase in LC_50_ value was not statistically significant. When comparing LC_50_ values between equal media, no statistically significant difference was found.

Rearing of *Artemia* cysts in unspiked media did not result in statistically different hatching rates between the different media (*p* = 0.715). The hatching rate recorded in the negative controls of the hatching success setup was 67.03 ± 1.75, 67.08 ± 2.45, 67.03 ± 1.60, and 70.78 ± 1.96% for ASW and DOM#1–3, respectively. Mercury coexposure in different media did not significantly affect hatching rates in comparison with single exposure in ASW (Table 2).

**Table 2:** Computed LC_50_ (µmol) and hatching EC_50_ (nmol) values after 24 h exposure of Instar I *Artemia* sp. nauplii and cysts to HgCl_2_ and HgOAc_2_

Media	LC_50_ (µM)		Hatching EC_50_ (nM)	
	HgCl_2_	HgOAc_2_	HgCl_2_	HgOAc_2_
ASW	**27.03** (17.80–36.26)	**24.59** (11.68–37.49)	**262** (239–285)	**255** (213–296)
DOM#1	**48.23** (31.67–64.79)	**62.89** (32.35–93.45)	**204** (139–269)	**196^** (175–216)
DOM#2	5.63*** (4.22–7.04)	8.65** (1.40–15.91)	**228** (176–279)	**324** (198–450)
DOM#3	2.59*** (1.77–3.40)	2.11*** (0.94–3.27)	**232** (113–350)	**225** (182–269)

Values are given as means (bold) of independent triplicates (95% confidence interval)

^*^Statistically significant values compared to ASW single exposure

^Values determined in duplicate

### Bioaccumulation assay

**Fig. 3:**
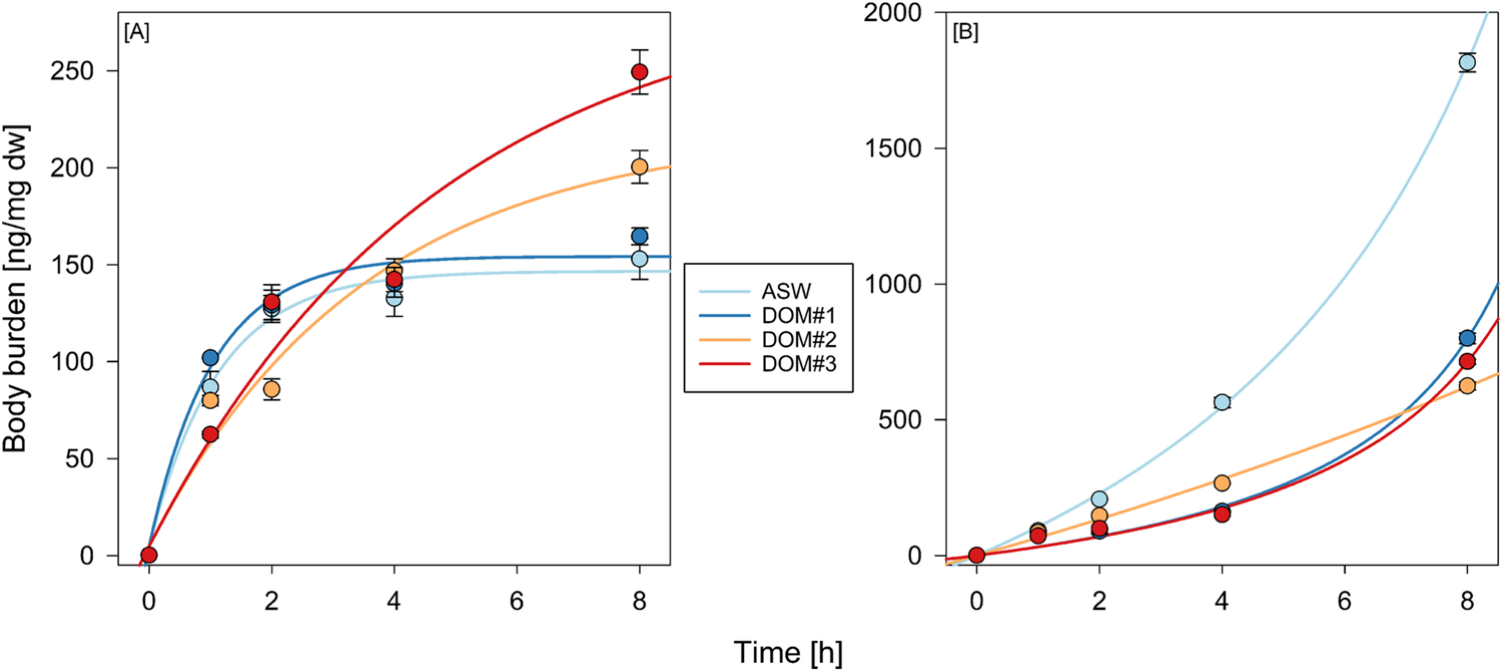
Mercury body burden measured in the **A** bioaccumulation assay and **B** the adsorption assay using Instar I nauplii of *Artemia* sp. Assays were conducted in independent triplicate. Solid lines denote **A** the one compartment accumulation model and **B** the non-linear pseudo-second order kinetic adsorption model

The increase in Hg body burden during exposure followed different patterns depending on media composition. In all cases, the fitted models provided a good description of the data (Fig. 3 and Table 3). Throughout the bioaccumulation assay, each treatment exhibited a substantial initial rise in concentration, which was subsequently followed by a period of stabilization. Pure ASW and DOM#1 coexposure resulted in lower final body burdens (153 and 165 ng/mg dry weight (dw), respectively) when compared with DOM#2 and DOM#3 coexposure treatments (200.4 and 249.3 ng/mg dw, respectively). For the ASW and DOM#1 coexposure group, an uptake plateau (no statistically significant body burden change) was reached after 2 and 4 h, respectively. In the case of pure ASW and DOM#1 coexposure, both accumulation and excretion rates were higher than those computed for DOM#2 and DOM#3 coexposure (Table 3). Further, the ratio of accumulation to excretion was greater for DOM#2 and DOM#3 coexposure resulting in overall lower elimination and higher body burden over time (Fig. 3). In the adsorption assay, all DOM coexposures led to generally decreased final body burdens in comparison with pure ASW media (− 60%). All adsorption data were characterized by a slow initial uptake followed by an exponential increase.

**Table 3:** Parameters derived from the one compartment bioaccumulation model (Eq. 1) and the pseudo-second order kinetic adsorption model (Eq. 2)

Media	Parameters					
	*k*_A_ (ng h^−1^)	*k*_E_ (h^−1^)	*k*_A_/*k*_E_	*r*^2^	k_2_ (mg g^−1^ min^−1^)	*r*^2^
ASW	125.48	0.88	141.94	0.99	1.76E − 03	1.00
DOM#1	143.72	0.96	149.56	0.98	5.53E − 03	0.99
DOM#2	61.09	0.28	215.11	0.97	6.59E − 04	0.99
DOM#3	61.33	0.21	290.65	0.96	5.23E − 03	0.99

## Discussion

### FTIR spectra of DOM compounds

DOM#1 has a unique band at 3692 cm^−1^, which, in combination with a peak at 913 cm^−1^, is commonly attributed to the hydroxyl groups of kaolinite, indicating an aluminosilicate contamination of the sample (Merlin et al. 2015). This is further supported by the peak at 1089 cm^−1^ which may be caused by Si–O stretching. The prominent peaks at 1563 and 1378 cm^−1^ are characteristic of carboxylic ions, with the latter typically being attributed to antisymmetric stretching of carboxylic groups (Stevenson and Goh 1971; Wu et al. 2016). Finally, the double peak at 1040–1020 cm^−1^ may be assigned to C-O stretching of polysaccharides or polysaccharide-like substances (Stevenson and Goh 1971; Machado et al. 2020).

The spectrum of DOM#2 has a unique peak at 1614 cm^−1^ associated with carbon double bond stretching in either aromatic, conjugated carbonyl, or carboxylate functional groups (Niu et al. 2019). The shoulder at 1720 cm^−1^ indicates the presence of small amounts of ketonic or aldehydic C = O groups (Stevenson and Goh 1971). Additionally, the spectrum has a unique additional peak at 1536 cm^−1^ attributed to amide functional groups or peptide linkage of proteins (Stevenson and Goh 1971). The bands at 1470 and 1430 cm^−1^ have previously been assigned to aliphatic C-H stretching and deformation (Wu et al. 2016; Machado et al. 2020). Tatzber et al. (2007) identified the small peak at 1260 cm^−1^ as either nitrate, = C–O–C groups, phenolic groups, or even P = O vibrations. The small peak at 1197 cm^−1^ may correspond to C-O stretching and O–H deformation of carboxylic groups or a contribution by aryl ethers (Stevenson and Goh 1971). The small bands at 1123 cm^−1^ and 1084 cm^−1^ indicate the presence of C-O bonds in esters, ethers, alcoholic, or phenolic groups (Niu et al. 2019), and C-O of alcoholic and aliphatic ethers, respectively (Tatzber et al. 2007). Similarly to the spectra of DOM#1, the group of low intensity bands between 1000 cm^−1^ and 1100 cm^−1^ may either be caused by Si–O stretching or C-O stretching of polysaccharides.

The spectrum of DOM#3 has a lower abundance of absorption bands and an overall lower absorption intensity. The most prominent peaks at 1560 and 1370 cm^−1^ are characteristic of carboxylic ions (Stevenson and Goh 1971), while the smaller peak at 1112 cm^−1^ is assigned to C-O bonds in esters, ethers, alcoholic, or phenolic groups (Niu et al. 2019). Similarly to the spectra of DOM#2, the small peak at 1034 cm^−1^ is attributed to C-O stretching of polysaccharide or polysaccharide-like substances (Stevenson and Goh 1971; Machado et al. 2020).

### Environmental relevance of Hg and DOM concentrations tested

Environmental levels of Hg in open marine ecosystems typically range between 0.8 and 2.5 pM (Gworek et al. 2016) depending on sampling depth and regional differences affecting, e.g., surface evasion (Zhang et al. 2023). Hotspots of marine water pollution can be found in semi-enclosed marine systems due to limited water exchange and proximity to anthropogenic pollution (Thongra-Ar and Parkpian 2002; Shadrin et al. 2021). Consequently, some of the highest concentrations measured outside of freshwater systems include lagoons (285 pM, Lacerda and Gonçalves 2001) and inland shelf seas (1670 pM, Shadrin et al. 2021). Like Hg, DOM and dissolved organic carbon (DOC) are typically found in lower concentrations in the marine environment than in freshwater environments (Del Vecchio and Blough 2004). Environmental DOC concentrations may range between 0.4 and 0.8 mg/l in the open ocean (Dittmar and Stubbins 2014), average 1.24 mg/l in coastal waters (Lønborg et al. 2024), and reach higher concentrations in semi-enclosed systems depending on regional parameters (Connolly et al. 2021; Amaral et al. 2023). According to an approximation by Krogh (1934), DOC makes up about half of DOM, which is reflected in the high carbon contents measured for DOM#2 and DOM#3 (Table 1).

The Hg concentrations used in this study are not environmentally relevant and were chosen to induce acute toxicity. The concentrations of DOC used in this study (~ 3–14 mg/l) fall within ranges reported for semi-enclosed marine systems, where *Artemia* are most prevalent. Further dilutions of DOM-enriched media were not carried out to observe the maximum impact of Hg-DOM coexposure.

### Acute toxicity and hatching assay

DOM#1 coexposure resulted in a non-significant decrease in toxicity in comparison with single exposure in ASW medium. DOM#2 coexposure significantly increased toxicity almost fourfold while DOM#3 coexposure increased toxicity more than tenfold. Humic substances are known to elicit cellular responses although the exact modes of action seem to be dependent on molecular characteristics. While some in vitro and in vivo studies report an induction of oxidative stress leading to a multitude of mutagenic and cytotoxic effects (Bernacchi et al. 1996; Cheng et al. 2003; Qi et al. 2008; Kihara et al. 2014), others suggest humic acid coexposure to alleviate heavy metal-induced oxidative stress by scavenging reactive oxygen species (Wang et al. 2021; Li et al. 2022). Oxidative stress caused by humic acids leads to a depletion of intracellular antioxidant levels including glutathione which also acts as an unspecific line of defence against heavy metal exposure (Qi et al. 2008; Krupp et al. 2016). Coexposure to DOM#3 may have induced an additive toxic impact, consequently causing a notable decrease in the derived LC_50_ values (Table 2). In comparison, the added polysaccharide moiety of DOM#1 (Fig. 1) may have inhibited an effective uptake of potentially toxic amounts of humic acid as it is most likely incapable of crossing lipid bilayer membranes. *Artemia* use DOM as a food source (Baylor and Sutcliffe 1963), which allows for the potential retention of Hg-DOM complexes within their gastrointestinal tract, thereby prolonging the time window to penetrate the epithelium. In the case of DOM#2, the abundance of functional groups and high sulfur content are ideal properties to effectively bind Hg. Additionally, alginate, the main constituent of DOM#2, is not known to elicit any toxicity which could have had an additive effect during coexposure.

**Fig. 4:**
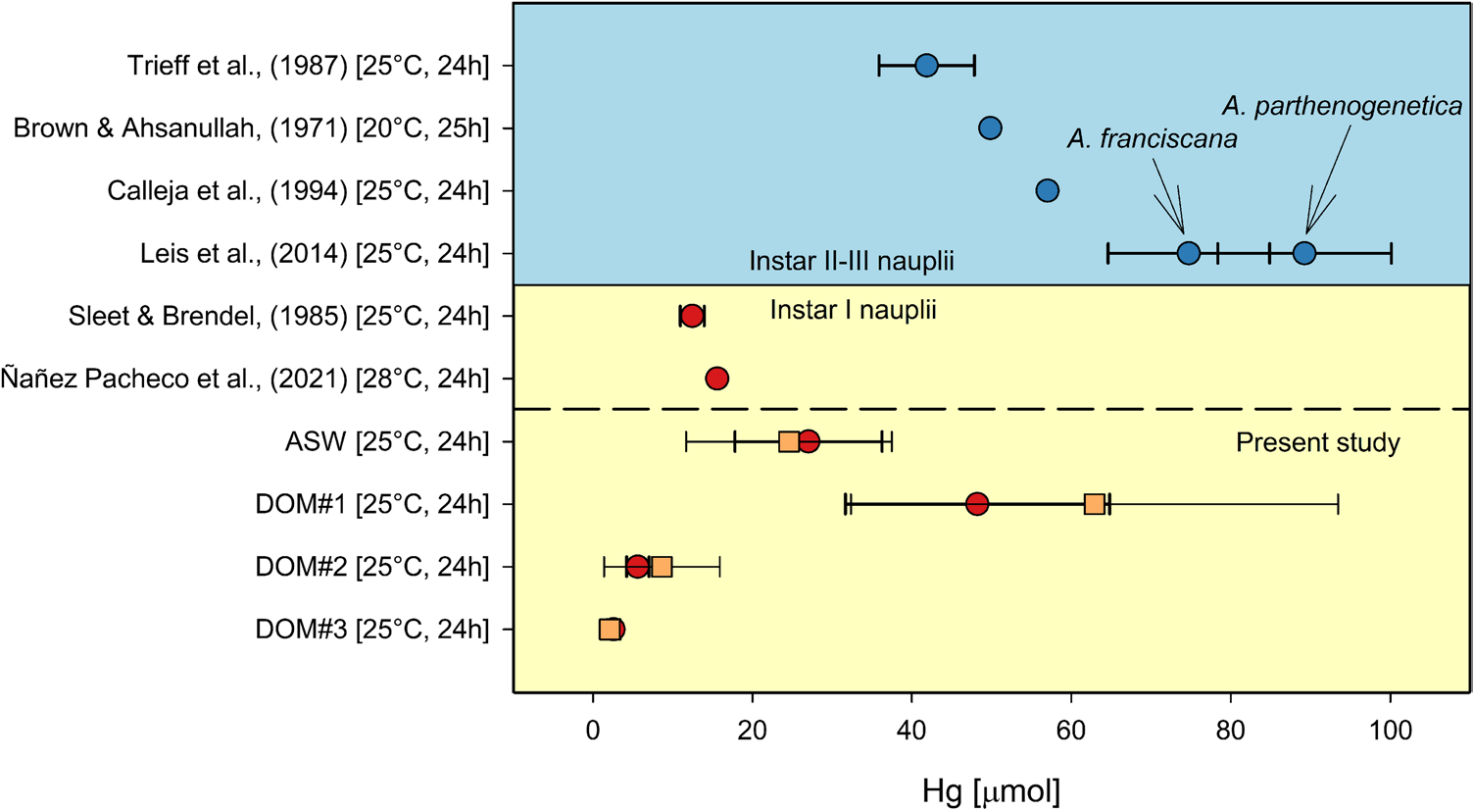
Literature 24 h LC_50_ values derived from *Artemia* Instar I (yellow half) and Instar II + III nauplii (blue half) after exposure to Hg. Values are given as mean and the error bars denote the 95% confidence interval if provided. Red and blue datapoints were derived from HgCl_2_ exposure, orange datapoints were derived with HgOAc_2_

A direct comparison of toxicological data derived in this study with existing literature is convoluted due to the use of non-standardized protocols in existing studies as well as this one. While both studies reporting LC_50_ data for Instar I nauplii used synthetic sea salts to prepare their exposure media (Sleet and Brendel 1985; Ñañez Pacheco et al. 2021), differences in their ionic composition are known to cause differences in metal toxicity (Arnold et al. 2007). Further, Ñañez Pacheco et al. (2021) conducted their experiments at 28 °C rather than 25 °C, while Sleet and Brendel (1985) conducted their experiments at an undefined exposure salinity, both of which have been reported to affect toxicity (Blust et al. 1992, 1994).

(Epi)genetic variability may also play a role, as strains differ in their susceptibility to Hg (Sarabia et al. 1998). Comparative studies assessing potential differences in toxicant resilience between different *Artemia* strains report contrasting results. While Leis et al. (2014) reported no significant difference in Hg toxicity between *franciscana* and *parthenogenetica* strains reared under laboratory conditions (Fig. 4), Pais-Costa et al. (2020) concluded that naturally occurring *Artemia* species may have adapted to locally elevated Hg concentrations, resulting in a significantly higher resilience. Similar results were obtained by Saliba and Krzyz (1976) who reported an increased tolerance of copper acclimated *Artemia.* Brown and Ahsanullah (1971) even reported an increased toxicity in A*rtemia* nauplii with decreasing Hg concentration which they could not explain. The LC_50_ values derived from the single exposure in ASW medium in this study fall within the range of previously reported data, although the susceptibility of the here used Artemia is slightly lower (Fig. 4).

The major complexing groups in marine waters are chloride and DOM with the latter being more spatiotemporally variable. Gebhardt (1976), Lavtizar et al. (2018), and Deese et al. (2016) reported a decrease in the toxicity of Hg, copper, and organic surfactants with elevated DOM concentrations. Penttinen et al. (1998), Lawrence and Mason (2001), and Day (1991) observed the same effects during their studies on the effect of DOM on the toxicity and bioaccumulation of cadmium, Hg, and synthetic pyrethroids in *Daphnia magna* and *Leptocheirus plumulosus*. Penttinen et al. (1998) further hypothesized that water hardness decreased the cadmium-DOM binding coefficient which may be due to cadmium being outcompeted by calcium ions. This is further supported by the works of Mantoura et al. (1978) who reported that more than 90% of Hg is chelated by humic acids in freshwaters, while in seawater more than 99% of humic substances are chelated by calcium and magnesium. This may explain the lack of impact of DOM coexposure on Hg toxicity in *Ceriodaphnia dubia* larvae reported by Mcnaughton (2007).

In contrast, Olivero-Verbel et al. (2008) determined the toxicity of landfill leachate to *Artemia* and reported that organic matter concentrations correlated positively with increasing toxicity. They hypothesized that apart from an inherent toxicity of uncharacterized organic compounds, their complexation of other inorganic elements may elicit an increased biological activity in comparison with free ions. Indeed, previous studies have observed increased toxicities due to organic complexation (Winner 1984; Winner and Gauss 1986), although this was highly dependent on the type of heavy metal used during exposure as has been noted by other researchers (Mcnaughton 2007).

Hatching success is time and temperature dependent and is more sensitive to Hg exposure relative to the acute toxicity assay with hatched organisms. The hatching success of approximately 70% at 25 °C following a 24-h incubation in the control group was consistent with findings from prior studies (Sorgeloos et al. 1978; Go et al. 1990). There was no statistically significant difference in toxicity between the different exposure groups in the hatching assay (Table 2). *Artemia* cysts are highly resistant to a range of physicochemical stressors due to embedded matrix peptides and the shells’ properties as a charge barrier (Dai et al. 2011). It is known that Hg speciates in saline matrices to form the stabilized tetrachlorido complex (HgCl_4_^2−^), which significantly decreases hatching toxicity by impeding cyst penetration (Okasako and Siegel 1980). The data reported by Wright and Mason (2000) further support the hypothesis that Hg uptake is not dependent on free ion concentration but rather amount and form of neutral Hg complexes in solution. The similarity between LC_50_ values in all exposure scenarios demonstrates that, although some DOM chelation occurred, the excess free Hg equally speciated to form charged chloride complexes which equally impacted their ability to cross membranes. A significant impact of chelation on hatching success was reported by Okasako and Siegel (1980) and Siegel et al. (1991) who used equimolar amounts of nucleophilic and chalcogenidic compounds. However, while the outer shell acts as a charge barrier, the underlying embryonic cuticle is permeable for lipophilic chemicals (Siegel et al. 1991; Patterson et al. 2021). Indeed, Go et al. (1990) reported that Hg entered *Artemia* cysts only after the outer shell had cracked, exposing the permeable embryonic cuticle. HgCl_2_ does not fully dissociate like other heavy metal salts and, due to having a low dipole moment, behaves more like a non-polar compound (Deng and Li 2022). This unique phenomenon enables HgCl_2_ to cross lipid bilayer membranes without requiring an active transport protein (Gutknecht 1981). Still, lipophilicity is a necessary but not sufficient condition for transport of Hg species across membranes. The reactivity with cell constituents determines the generation of a concentration gradient and thus the direction of diffusion. Mason et al. (1996) and Braeckman et al. (1998) were able to demonstrate that inorganic Hg targets inner cellular membranes resulting in a constant diffusion into cells.

**Fig. 5:**
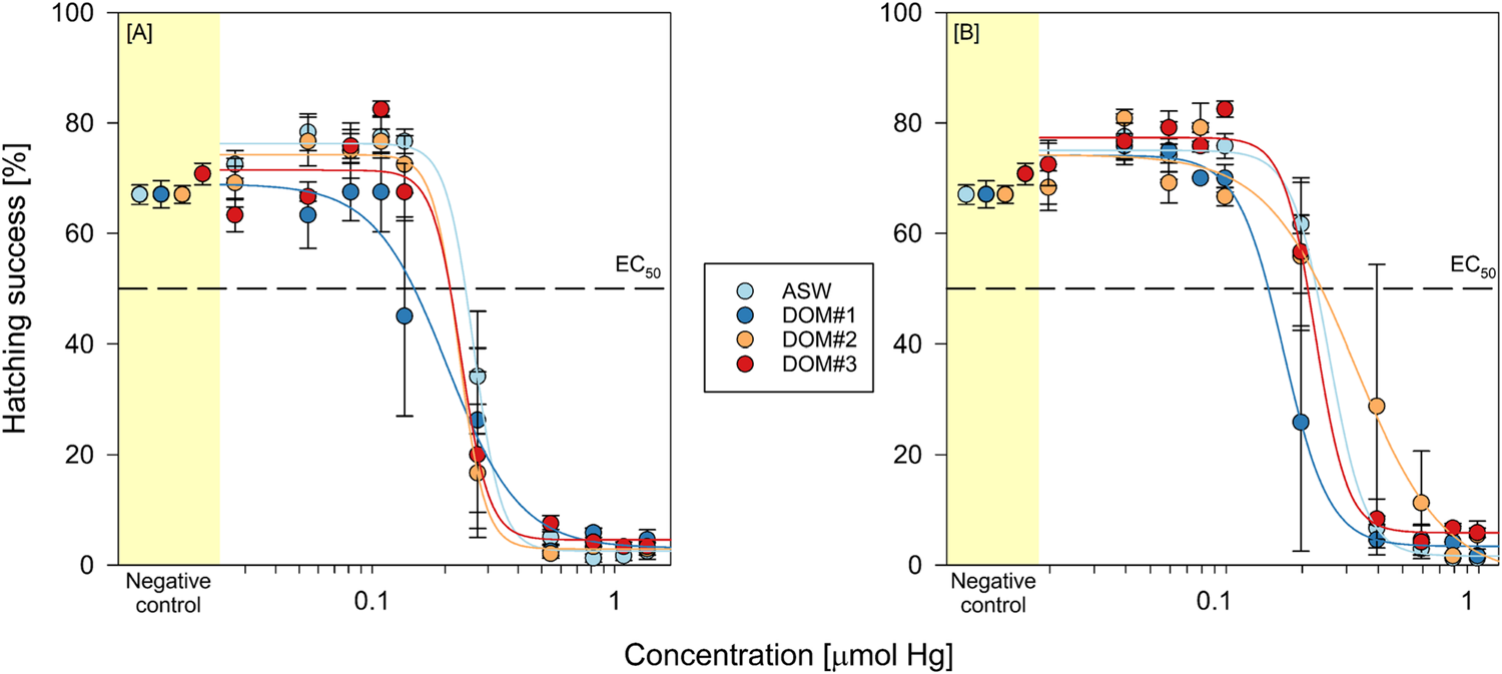
Hatching success of *Artemia* sp. cysts after 24 h coexposure to **A** HgCl_2_ and **B** HgOAc_2_ with DOM#1–3 media (see respective colors). Assays were conducted in independent triplicate, solid lines show the combined four parameter logistic fit, and error bars denote the standard error of the mean

It is difficult to compare the here produced results with previous hatching studies as reported EC_50_ values are either an order of magnitude higher (100 mg/l, Liu and Chen 1987) or were never determined due to and inadequate choice of concentration range (Ñañez Pacheco et al. 2021). It should be noted that the hatching process did not follow a proportional dose–response curve but rather exhibited a threshold concentration at which the hatching success pivoted (Fig. 5). So far, Go et al. (1990) have provided the most comprehensive explanation of the effect of Hg on *Artemia* hatching. They hypothesized that Hg affects the Na,K-ATPase in the larval salt gland, resulting in a disruption of the required osmotic potential to break the inner cuticle., Hg inhibits multiple transmembrane ion exchange mechanisms through non-competitive inhibition of membrane transport ligands (Wright and Welbourn 1991; Anner et al. 1992). Furthermore, Hg is known to affect the osmoregulation capacity in various crustaceans (Lignot et al. 2000), which supports the hypothesis of Go et al. (1990).

*Artemia* have been recommended for use in routine ecotoxicological testing due to their easy procurement and convenient deployment (Wisely and Blick 1967), leading to the development of additional endpoint assays (Libralato et al. 2016). However, *Artemia* are uniquely resistant to metals (in the case of Hg by a factor > 300), in comparison with other marine and freshwater organism assays (Calleja et al. 1994; Kalčíková et al. 2012). Mercury still affects *Artemia* at sublethal concentration as documented in phototaxy studies by Palmer Saunders et al. (1985) and Trieff et al. (1987), who reported an increased photomotility during low level Hg exposure. Sarabia et al. (1998), and more recently, Kim et al. (2020) hypothesize that this may be caused by a general hormesis effect of Hg exposure on *Artemia* nauplii. However, contrasting studies by Yaeger et al. (1986) and Siegel et al. (1991) showed that early-stage nauplii exhibited a decreased phototaxy at concentrations as low as 0.1–1 µM and put emphasis on the importance of developmental stage during the exposure period as pointed out by other researchers (Vanhaecke and Persoone 1984). Indeed, *Artemia* exhibit an increasing susceptibility with progressing development (Fig. 4, Kalčíková et al. 2012), originally explained by the absence of fluid ingestion and the subsequent protection of the digestive tract epithelium (Sorgeloos et al. 1978). However, later works by Sleet and Brendel (1985) investigating the underlying reasons for this progression suggested that Hg targets specific moulting processes. Cotou et al. (2001) were able to identify ATPase inhibition as a dominant factor in perturbing naupliar stage advancement thus further supporting a development stage dependent toxicant susceptibility.

### Adsorption and bioaccumulation assay

DOM complexation is known to decrease Hg diffusivity through chelation (Gade et al. 2024), which may explain the significantly lower adsorption rates in DOM coexposures in comparison with pure ASW. The rate limiting step in this experiment is the chemisorption onto the adsorbate which is considered in the herein used pseudo-second-order kinetic model. The experimental data does not follow the typical trend of adsorption data (exponential rise to a maximum), thus making a comparison difficult. Similar Hg adsorption data were produced by Lopes et al. (2003) and Cestari et al. (2004), who used the Avrami kinetic model to describe their sigmoid values (Avrami 1939). However, the use of the Avrami kinetic model outside its intended purpose, including adsorption studies, is disputed as it was originally developed to describe phase changes in materials science (Oladoja 2016; Lima et al. 2016; Shirzad and Viney 2023).

Bioaccumulation studies are pivotal in understanding analyte behaviour in complex food webs. *Artemia* have been observed to thrive in Hg contaminated habitats and possess the concentrate it by a factor of 100 over ambient concentrations (Liu and Chen 1987; Naftz et al. 2008; Wright et al. 2020). As previously mentioned, the combined effect of coexposure to DOM and the potential retention of DOM complexes in the gastrointestinal tract may adversely affect the organism's ability to eliminate toxins, allowing complexed Hg to accumulate and penetrate the epithelium over time. Initial studies on the accumulation of Hg compounds in *Artemia* proposed that their high resistance to Hg exposure could be attributed to a slow uptake rate (Corner and Rigler 1958). However, the data generated in this study suggest that, instead, an unobstructed depuration mechanism results in a decrease and eventual plateau in Hg uptake. Similar data were reported by Corner and Rigler (1958) and Liu and Chen (1987) in their investigations of Hg accumulation in *Artemia*, as well as by Heugens et al. (2003) in their study of cadmium accumulation in *Daphnia magna*, indicating a shared physiological depuration process. Saxton et al. (2013) demonstrated that the transfer of maternal Hg into cysts is restricted, which serves as a crucial safeguard since exposure to concentrations as low as 10 nM may impede organism development (Go et al. 1990). Organisms that do hatch after low level exposure show signs of abnormal segmentation, reduced size at hatching, and a hormetic effect on the growth rate as well as a significantly shorter lifespan (Go et al. 1990; Sarabia et al. 1998).

### Environmental implications

Acute Hg toxicity is typically not considered a risk in environmental pollution research as natural levels are insufficient. However, uptake by benthic biota and biomagnification along the food chain are considered important factors in environment risk assessment (von Hellfeld et al. 2023b). Filter feeding crustaceans such as Artemia serve as a springboard for pollutants into larger food webs such as waterfowl (Jones and Wurtsbaugh 2014; Shadrin et al. 2022). As such, Hg chelation by naturally occurring DOM may increase overall bioaccumulation rates, depending on their molecular makeup. In a comparative study, Schartup et al. (2015) compared the effects of marine and terrestrially derived DOM on aquatic Hg speciation and bioavailability. They concluded that marine DOM seems to facilitate cellular uptake of Hg, which in turn may influence bioaccumulation or benthic methylation rates. Due to its spatiotemporal variability, DOM remains an important dynamic factor in contaminant cycling and may affect uptake rates and patterns.

## Conclusion

Despite the lack in standardization concerning assay parameters and media composition, microcrustaceans, including *Artemia,* remain a quick and simple in vivo test system for high-throughput toxicity testing with the potential to be an asset in future routine screening. This work highlights the profound impact of diverse types of DOM on the toxicity of Hg, which so far remains underexplored in ecotoxicity testing. While DOM coexposure did have a significant impact on hatched organisms, encysted *Artemia* sp. are not affected by varying amounts of DOM in the surrounding medium. Our results demonstrate that a definitive statement on DOM-Hg interactions cannot be made as the type of DOM is directly affecting Hg toxicity. We recommend future studies to investigate the DOM-mediated toxicity and bioavailability for other metals, with special focus on the chemical composition of the organic ligands and their functional groups.

## Data Availability

Data will be made available on reasonable request.
